# Descriptive analysis of hypertension in Saudi Arabia: a national population health perspective (hypertension in Saudi Arabia)

**DOI:** 10.3389/fcvm.2026.1758741

**Published:** 2026-03-26

**Authors:** Malak E. Aloufi, Mohammed AlJawadi, Yahya A. AlMazni, Abdulrahman Alsheikh, Abdulaziz Eskandarani, Abrar Alasmari, Mubarak Aldawsari, Shatha Alshaibi, Aisha M. Alshehri, Meral Homoud, Shaker A. Alomary, Mariam M. Al Eissa, Abdullah Assiri, Mohammed Abdulali

**Affiliations:** 1 Population Health Observatory, Ministry of Health, Riyadh, Saudi Arabia; 2 Benefits and Outcomes Department, Center of National Health Insurance, Riyadh, Saudi Arabia; 3 Population Health Department, Lean Business Services, Riyadh, Saudi Arabia; 4 College of Medicine, Alfaisal University, Riyadh, Saudi Arabia; 5 Public Health Lab, Public Health Authority, Riyadh, Saudi Arabia; 6 Research Department, King Khaled Eye Specialist Hospital (KKESH) Research Centre, Riyadh, Saudi Arabia; 7 Computational Sciences Department at the Centre for Genomic Medicine (CGM), King Faisal Specialist Hospital & Research Center, Riyadh, Saudi Arabia

**Keywords:** Saudi Vision 2030, electronic health records, epidemiology, health services, hypertension, obesity, population health observatory (PHO)

## Abstract

**Background:**

Hypertension (HTN) is one of the most significant public health challenges in Saudi Arabia, driven by ageing, lifestyle changes and rising metabolic risk factors. Although the prevalence of HTN is increasing in Saudi Arabia, there have been limited studies and updates of national-level numbers.

**Objective:**

This study provides a national-level descriptive overview of high blood pressure in Saudi Arabia using data from the Population Health Observatory (PHO). These data include prevalence, demographics and characteristics, comorbidities and risk and healthcare utilisation.

**Methods:**

A retrospective observational study using data from the PHO from 2015 to 2025 was conducted, including all 1.7 million diagnosed individuals across all regions of Saudi Arabia. Descriptive analyses were performed for the variables.

**Results:**

The analysis revealed 1,720,786 hypertensive patients in Saudi Arabia. The highest burden falls on males, accounting for 58.2% of hypertensive patients. Of those males, the majority were aged 50–59 years. Most patients were obese or overweight (29.4% and 21.8%, respectively). The majority of the patients had a rising risk profile, and most of them visited outpatient clinics (88.9%).

**Conclusion:**

HTN remains a major public health concern in Saudi Arabia. The data from the PHO highlighted clear results and patterns that can guide interventions and policymakers in strengthening prevention and improving health promotion and control.

## Introduction

Worldwide, high blood pressure (HBP) is one of the biggest risk factors for death and disability (1). The prevalence of hypertension (HTN) increased between 1990 and 2019, from 650 million to 1.3 billion (1, 2). This disease, which includes HTN and hypertensive heart disease (HHD), with its increasing prevalence, has become a major focus of public health authorities. It affects approximately 18.6 million cases, and chronic kidney disease (CKD), and rose by 162% during the same period (1, 3, 4). In addition, the disease causes various musculoskeletal conditions, including osteoarthritis (5, 6). Due to urbanisation, physical inactivity and an unhealthy diet, it is expected that the prevalence of HTN will increase both locally and worldwide (7). In addition, the prevalence of HTN increases with life expectancy, especially in elderly persons (8). To reduce the burden of non-communicable diseases, the Kingdom has implemented a healthcare transformation plan within Saudi Vision 2030. A core component of this transformation is to shift from treating diseases to prioritising prevention and promoting long-term health (6). A major aim of this transformation is to address HTN because of its high prevalence and modifiable risk factors (6). Although some studies have looked at the national and regional frequencies of HTN in Saudi Arabia, these studies might have some limitations (9, 10). Only three studies looked at the prevalence of HTN in Saudi Arabia, one of which was a regional-level study conducted between 1989 and 1994 (9), while another was conducted nationwide (10). However, the nationwide and regional prevalence rates of HTN have not been reported or addressed in any of those studies. A third study was recently published on the prevalence and risk factors of HTN in Saudi Arabia. The nationally representative survey revealed a crude prevalence of 12.7% among adults aged 18 years and older and an age-standardised prevalence of approximately 22.3% (11). Therefore, the evidence about HTN in Saudi Arabia has neither been updated nor does it include the whole population; thus, it may not represent the real-world prevalence of HTN (12). This emphasises the need for an updated study and revision of the current data on HTN prevalence. Although previous national and regional studies exist, some gaps remain in the understanding of HTN in Saudi Arabia. The previous studies are either outdated or limited to a specific region, and they do not provide comprehensive national prevalence stratified by age, sex, region, insurance status and other demographic characteristics. Furthermore, limited evidence is available regarding the patterns of comorbidities and healthcare utilisation among the population with HTN at the national level. Accordingly, this study aims to answer the following research questions: What is the current national prevalence of HTN among adults in the Kingdom of Saudi Arabia? How is HTN distributed across demographic and geographic groups, including age, sex, nationality and region? What are the patterns of comorbidities and healthcare utilisation among the HTN population? The Population Health Observatory (PHO) under the Population Health Department of the Saudi Ministry of Health aimed at strengthening the Kingdom's public health infrastructure through an improved data repository for surveillance, disease burden monitoring and strategic policy guidance. In line with Saudi Vision 2030, the PHO addresses population-level health challenges and is poised to serve as a national platform for health intelligence (13–15).

The PHO combines various health data sources and advanced data integrative techniques, enabling comprehensive surveillance beyond mere signal detection (14). It has enhanced surveillance capabilities by including social determinants of health (SDOH) that impact population health outcomes. Therefore, this study aims to reveal the national real number prevalence of HTN in Saudi Arabia using integrated data from the population health observatory. In doing so, we will provide a descriptive overview on a population level, assessing the epidemiological and clinical characteristics by age, sex and region, focusing on the prevalence and distribution of HTN by demographic and geographic factors. Then we will evaluate the comorbidity patterns and risk stratification of patients and analyse the patterns of healthcare utilisation among HTN patients. Finally, we will provide evidence-based insights to guide public health interventions and policy development. The main purpose was to identify key trends that support the health system strategy and planning.

## Methodology

### Study design

In this study, the total sample size was 1,720,786. We performed a retrospective population-based observational design analysis from a dataset of HTN-related healthcare through the PHO at the MOH, where nationwide data are recorded. This research focuses on evaluating HTN among adults across the nation. Demographic and health-related information was extracted from the national population registry, including disease and death registries, with the risk factors, distribution, comorbidities, cost impact and service utilisation. Data were obtained from electronic health records (EHRs) covering all Ministry of Health primary healthcare centres (PHCCs) and both public and private hospitals in Saudi Arabia, utilising data from the Ministry of Health data bank, which incorporates Raqeem, Nphies, Wasfaty and the National Data Bank. The data contain dependent and independent variables, as follows: (A) Demographic characteristics: age (categorical groups), gender (male/female), nationality (Saudi/non-Saudi), residence (urban/rural), vital status (alive/dead), insurance status (insured/uninsured). (B) Clinical characteristics, including body mass index (BMI) categories (obese, overweight, healthy weight, underweight), blood pressure trends (e.g., fluctuating, stage 1, stage 2, elevated, crisis, etc.), and comorbidity risk stratification (rising risk, high risk, critical risk). Finally, (C) Healthcare utilisation: outpatient visits, emergency room visits and hospital admissions.

### Cohort definition and data sources

A national-level integrated health database was utilised to extract data from the PHO covering the period between 2015 and 2025, encompassing over 1.7 million individual health records. Individuals were identified using ICD-10 diagnostic codes related to HTN, including primary (essential) HTN (I10), secondary HTN (I15) (15), elevated blood pressure without a diagnosis of HTN (R03.0), HTN urgency (I16.0), HTN emergency (I16.1) and HTN crisis (I16.9). BMI was calculated as weight in kilograms divided by height in meters squared (kg/m²). BMI categories were defined according to the World Health Organization classification as underweight (<18.5 kg/m²), normal weight (18.5–24.9 kg/m²), overweight (25.0–29.9 kg/m²), and obese (≥30.0 kg/m²) (3, 16). Only individuals aged 18 years and above with at least one recorded HTN-related ICD-10 code were included. Records with incomplete demographic or diagnostic information were excluded. Longitudinal patient-level data were extracted, including demographics (age, sex and region), clinical indicators (BMI categories and blood-pressure patterns), hospital capacity, medication history and multimorbidity profiles such as diabetes and hyperlipidaemia, adapted to the local epidemiological context using the Charlson Comorbidity Index (CCI) (17).

### Data processing

To harmonise data entries, a pipeline was used to standardise data processing across health clusters. Multiple imputation techniques were used to address missing values for duplicated patients. IDs were cross-validated using national health data, and the incomplete health records were labelled and excluded from sub-analyses.

### Ethical approval and considerations

Ethical approval was obtained from the Central Institutional Review Board at the Ministry of Health ethics committee, Saudi Arabia, approval number 25–108 M, with National Registry Number with NCBE-KACST, KSA: (H-01-R-009). The data used in this study were anonymised, with the absence of identifiable patient information, and secondary data were obtained through the PHO portal in alignment with the data centre at MOH, in compliance with national data governance standards.

### Geospatial analytics

To visualise the incidence of the disease and mortality across the Kingdom and evaluate the regional burden, GIS-based heat mapping was utilised (15).

### Statistical analysis

National-level data were queried and processed using SQL in the Apache Impala Cloudera distributed environment, allowing us to analyse real-time data. Confidence intervals (CIs) were calculated using IBM SPSS Statistics version 26.0 (IBM Corp., Armonk, NY, USA). Excel tables and graphical representations were created to summarise the distribution of HTN cases across age groups, sex and regions. No advanced inferential or multivariable statistical modelling was applied, as the study aimed to provide a population-level descriptive overview.

## Results

A total of 1,720,786 individuals were identified as having HTN in the Kingdom of Saudi Arabia. Based on an adult population of 26,454,318 in 2025, this corresponds to an overall prevalence of 6.50%. More than half of the HTN individuals were male (58.20%, *n* = 1,001,953), while females accounted for 41.80% (*n* = 718,833). The majority were Saudi nationals (68.10%, *n* = 1,172,048), with non-Saudis representing 31.90% (*n* = 548,738). In terms of insurance status, 68.11% (*n* = 1,172,018) were uninsured and 31.90% (*n* = 548,768) were insured. Nearly all individuals resided in urban areas (99.86%, *n* = 1,718,460), whereas only 1.14% (*n* = 2,326) lived in rural areas. At the time of data extraction, 95.34% (*n* = 1,640,647) were alive and 4.66% (*n* = 80,139) were deceased (Table 1).

**Table 1:** Demographic characteristics of HTN patients in Saudi Arabia.

Characteristic	Category	Number	Percent of hypertensive (%)	Percent of KSA adults (%)	Percent of KSA adults by category (%)	95% CI (%)
Gender	Male	1,001,953	58.20	3.79	5.92	3.78–3.80
	Female	718,833	41.80	2.72	7.55	2.72–2.73
Nationality	Saudi	1,172,048	68.10	4.43	8.91	4.42–4.44
	Non-Saudi	548,738	31.90	2.07	4.13	2.07–2.08
Insurance state	Insured	548,768	31.90	2.07	6.73	2.07–2.08
	Uninsured	1,172,018	68.11	4.43	6.40	4.42–4.44
Residence	Urban	1,718,460	99.86	6.50	6.51	6.49–6.51
	Rural	2,326	1.14	0.01	3.17	0.01–0.01
Vital state	Alive	1,640,647	95.34	6.20	6.38	6.19–6.21
	Dead	80,139	4.66	0.30	10.84	0.29–0.30

**Figure 1:**
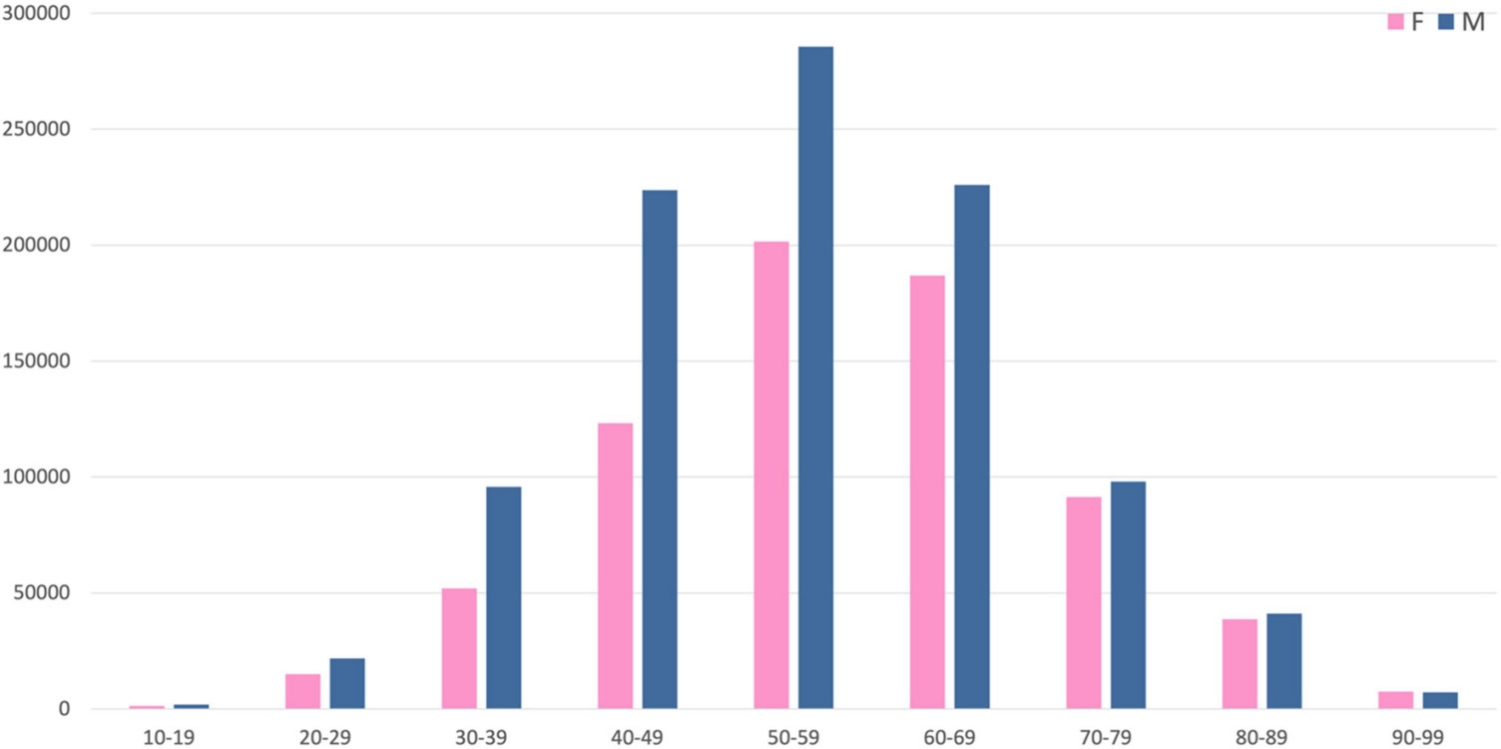
Distribution of hypertension cases by age group and gender.

Across age groups, as shown in Figure 1, the highest proportion of HTN cases was observed among individuals aged 50–59 years, accounting for 28.31% of hypertensive patients. Among HTN patients aged 50–59, the burden is greater in males (16.60%) compared to females (11.71%).

**Figure 2:**
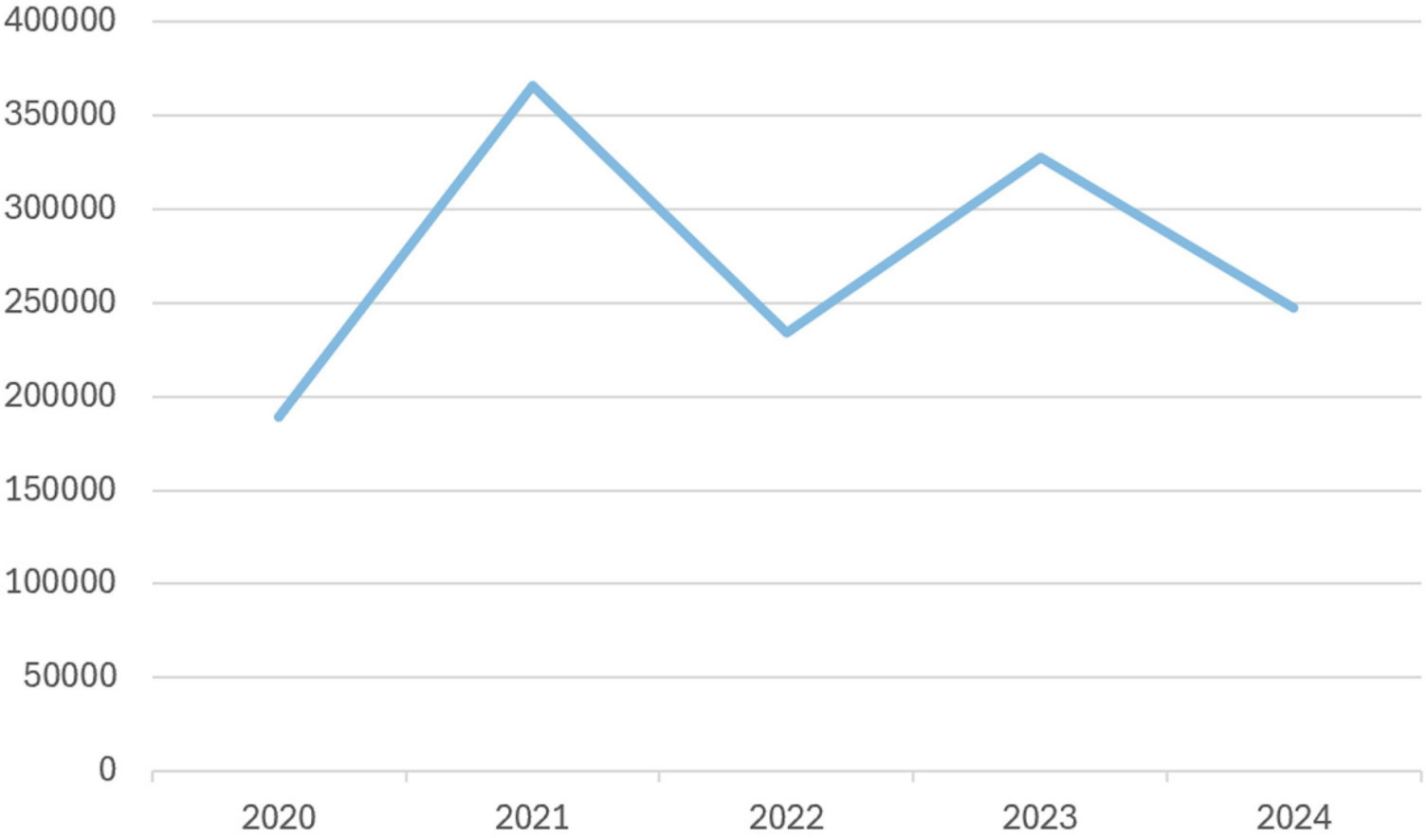
Incidence report of HTN diagnosis (2020–2024).

Across age groups, as shown in Figure 2, the incidence of hypertension diagnosis peaked in the year 2021. The distribution of hypertension patients according to BMI categories and blood pressure trends at least 1 month apart. Comorbidity risk stratification among HTN patients and healthcare utilisation among HTN patients are shown in Table 2.

**Table 2:** Body mass index (BMI) categories, comorbidity risk stratification and healthcare utilisation.

Category	Subcategory	Number	Percent of hypertensive (%)	Percent of all KSA adults (%)	Percent of KSA adults by category (%)
BMI category	Obese	506,422	29.43	1.91	15.81
	Overweight	374,774	21.78	1.42	10.94
	Healthy weight	219,286	12.74	0.83	3.58
	Underweight	8,746	0.51	0.03	0.19
Risk category	Rising risk	987,024	57.36	3.73	69.55
	High risk	129,586	7.53	0.49	110.78
	Critical risk	6,783	0.39	0.03	95.54
Type of visit	Emergency room visits	3,731,470	9.21	1.28	8.39
	Outpatient visits	35,974,427	88.81	12.37	15.07
	Hospital admissions	802,555	1.98	0.28	10.47

**Figure 3:**
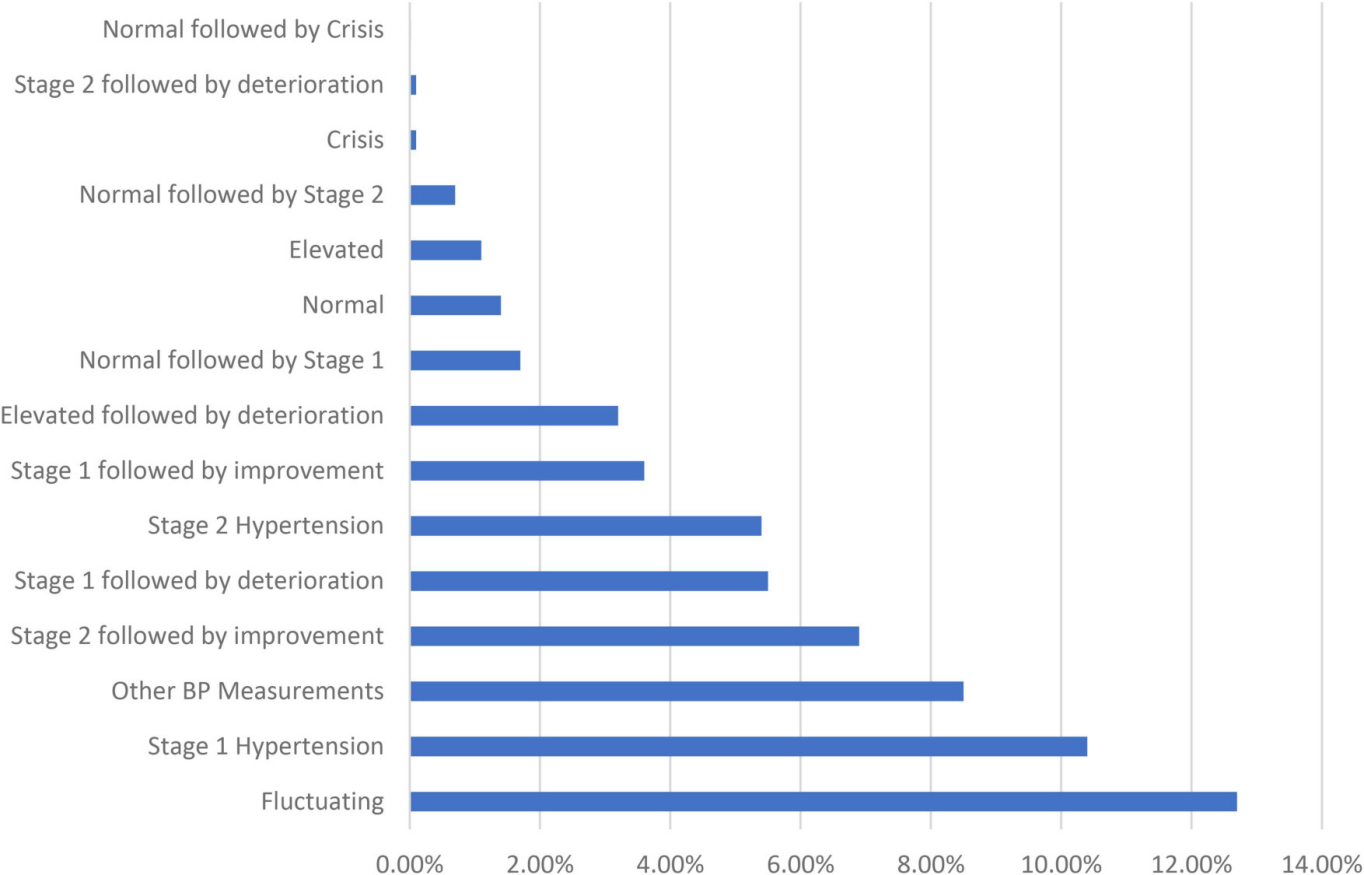
Blood pressure trends at least 1 month apart.

Among the blood pressure trends measured at least 1 month apart, the highest proportion was observed in the fluctuating category, accounting for 12.70% of hypertensive patients, while those without any blood pressure trends (37.30%) have not been included. The label “Other BP Measurements” refers to those that only have two readings at least a month apart, rather than the three needed for our classification criteria (Figure 3).

**Figure 4:**
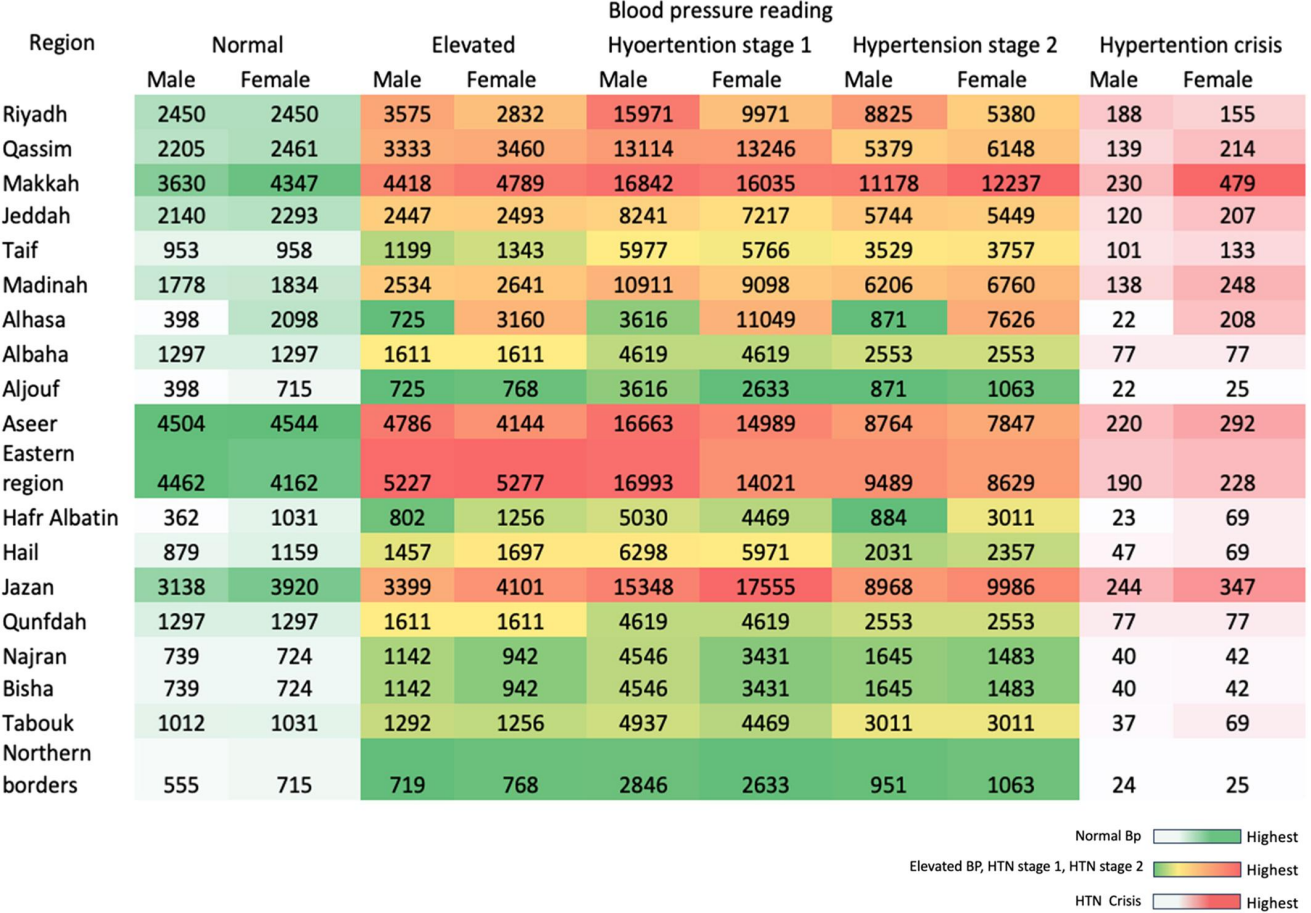
Distribution of hypertension patients in 2025 across regions. CDC Hypertension Stages & Categories. Normal: <120/80 mmHg. Elevated: 120–129 and <80 mmHg. Stage 1 Hypertension: 130–139 or 80–89 mmHg. Stage 2 Hypertension: ≥140 or ≥90 mmHg. Hypertensive Crisis: >180 and/or >120 mmHg. Source: CDC (24). 
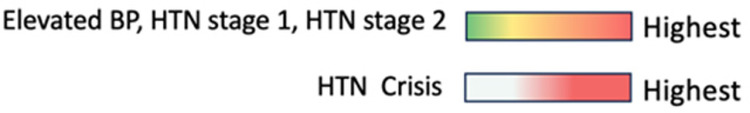

In examining the distribution of blood pressure categories in 2025 across regions and genders, clear patterns emerge. For HTN stage 1, males carried the largest burden, with 189,673 individuals, while females had 173,597 cases. For HTN stage 2 and HTN crisis cases, females had the highest frequencies overall, at 102,785 and 3,310, respectively. In contrast, the males presented with 99,391 cases for HTN stage 2 and 2,249 cases for HTN crisis. At the regional level, Jazan had the highest number of HTN stage 1 cases, with 32,903 individuals. For HTN stage 2 and HTN crisis, Makkah had the majority, at 23,415 and 809 cases, respectively. As for the region–gender cross section, the burden of stage 1 HTN was most pronounced among females in Jazan, with 17,555 cases. For HTN stage 2, the highest frequencies were again concentrated in Jazan, with females recording 9,986 cases. With respect to hypertension crisis, the most affected groups were females in Makkah with 479 cases (Figure 4).

## Discussion

HTN represents a substantial burden for the Saudi population; this national-scale descriptive analysis across the PHO dataset identified more than 1.7 million affected individuals. Its predominant effect was found among males with a 58.2% representation and a middle-aged peak prevalence (50–59 years). The higher proportion of HTN cases observed among individuals aged 50–59 years may be explained by the fact that this age group represents a transitional period during which long-term exposure to risk factors, such as obesity, diabetes, physical inactivity and unhealthy diet, begins to manifest clinically, in addition to age-related vascular changes. This pattern is consistent with previous national and international evidence showing that hypertension prevalence increases steadily with age.

The observed peak in HTN incidence in 2021 may reflect improved detection and healthcare engagement during that period, potentially influenced by improvements in the electronic health system after the COVID-19 pandemic. Blood pressure-related morbidity increases may be attributable to sedentary lifestyles, urbanisation and ageing populations (1, 4, 17). A recent WHO report, *Country Profile for Saudi Arabia (2023)*, showed that the standardised HTN prevalence registers at 33% in men and 29% in women among adults aged 30–79 years, which requires attention through national preventive programs (16).

Epidemiological determinants are associated with modernisation in urban regions with high concentrations of cases of vascular disease, based on 99.9% of the data. This can be explained by behavioural risk factors that are major contributors to HTN, derived from limited engagement in physical activity, experiencing higher psychosocial stress and consuming diets that are rich in sodium and energy-dense (7, 18). Our data revealed that among HTN patients, 29.4% were obese and 21.8% overweight, which aligns with previous findings through a national survey by Alenazi and Alqahtani (2023), which demonstrated a strong association with obesity and diabetes in 9.2% of HTN patients (19). Another local study by Nasser et al. (2025) identified the key predictors of 11.1% of hypertensive Riyadh adults as associated with advanced age, obesity and high cholesterol (18). This finding confirms that clustering metabolic risk factors is strongly linked to the epidemiology of HTN in the Kingdom. Recently, a study was published that revealed a crude HTN prevalence of 12.7% and an age-standardised prevalence of approximately 22.3%, with strong associations with older age, obesity, diabetes, dyslipidaemia and smoking (11). Regional variability was notably marked for stages 1 and 2 HTN; most HTN crises were detected in the Makkah region. These variations probably reflect heterogeneity in social determinants of health (SDOH), dietary patterns and access to primary care. However, previous research groups have highlighted regional discrepancies (9, 10). As an example, the crisis stage in Makkah was identified among females in a certain cluster, which has been reported before in a study by Alshammari et al. (2023), indicating a lower control showing a 35% and 42.8% awareness among females towards males. This difference can be controlled by targeted screening and a tailored awareness program (20). With those previous findings in the PHO, we confirm that real-time updated monitoring describes the post-Saudi Vision 2030 update at a high resolution at the population level.

### Health-system utilisation and preventive gaps

Outpatient service utilisation, reflecting the central role of primary-care management encounters, accounted for nearly 89%, with two-thirds of the lack of continuity of care, insurance and medication adherence. A study performed by Alruwaili (2024) indicated an urgent need for follow-up systems and strengthening health literacy to improve knowledge and adherence (21). It is crucial to embed HTN management to achieve sustained control and equitable coverage within the Saudi Vision 2030 Health Sector Transformation Program (22), because population-level surveillance and the longitudinal integration of electronic health records enabled by AI analytics provides a robust foundation for precision prevention (14).

In a comparative global context, the prevalence of HTN increased from 1990 to 2019 from 650 million to 1.3 billion (1, 4). A recent study mirrors this global trajectory by estimating a prevalence of 22.7% among individuals aged 14–100 years of age (95% CI 18.9–26.6) out of 278,000 participants (20). Furthermore, the coexistence of HTN with dyslipidaemia, diabetes and osteoarthritis, indicates a growing economic burden with the complexity of multimorbidity (6). However, this can be overcome with the implementation of a large-scale, data-driven program to prevent HTN-related cardiovascular and renal disease (23).

This descriptive finding reinforces the imperative of data-driven models to pivot from providing healthcare to preventive care. In the integration of SDOH, advanced AI analytics enable predictive risk modelling and equitable resource allocation (14). There is a plan to use geospatial dashboards to assess community-level screening and provide burden estimates nationwide, supporting the Kingdom's ambition for personalised, preventive healthcare.

One limitation of this study is the descriptive design and its reliance on administrative data, which limits further interpretation and may underrepresent other sectors, such as military and private-sector patients. Nonetheless, the national coverage that is provided by the PHO dataset provides data harmonisation to ensure representative, real-time, policy-relevant insights.

Future research should incorporate biological, multivariable data and incorporate with geospatial modelling to forecast long-term health-economic impacts and identify regional drivers of poor control. This study has some limitations. The PHO was only established in 2023, as described by Al Eissa et al. (2026), and therefore the integration and completeness of historical records may vary across data sources and regions. Also, as the study relies on routinely collected administrative and electronic health record data, some variables may be incomplete or subject to documentation errors, including potential misclassification or missing clinical information due to incorrect or inconsistent physician data entry. Last, it is not real-time data.

In conclusion, HTN remains a critical, modifiable public-health challenge in Saudi Arabia, influenced by behavioural and metabolic factors, as well as SDOH. Strengthening national coverage, facilitating patient awareness and leveraging PHO's data-integration capabilities are vital to reducing the cardiovascular morbidity burden, in alignment with population-health transformation as part of Saudi Vision 2030.

## Data Availability

The raw data supporting the conclusions of this article will be made available by the authors, without undue reservation.
